# Severe Pediatric Erythromelalgia: A Case Report on Multimodal Pain Management and the Role of Regional Anesthesia

**DOI:** 10.7759/cureus.71351

**Published:** 2024-10-13

**Authors:** Adil Zyani, Mouncif El Moutawakil El Alami, Rajae Alkouh, Ounci Es-Saad, Smael Labib

**Affiliations:** 1 Critical Care Medicine, Mohamed VI University Hospital, Tangier, MAR; 2 Intensive Care Unit, Mohamed VI University Hospital, Tangier, MAR; 3 Anesthesiology and Reanimation, Mohamed VI University Hospital, Tangier, MAR

**Keywords:** chronic and acute pain management, neuropathy, primary erythromelalgia, regional anesthesia and chronic pain, vasculopathy

## Abstract

Erythromelalgia is a rare neurovascular condition characterized by episodic burning pain, erythema, and increased temperature of the extremities. This condition, particularly challenging in pediatric patients due to its rarity and the complexities of pain management, often results in significant distress and impaired quality of life.

We report the case of a 14-year-old patient who presented with severe primary erythromelalgia. Despite extensive pharmacologic interventions, the patient's pain was refractory, leading to significant psychological distress and a suicide attempt. The pain was eventually managed with carbamazepine, bilateral sciatic nerve blocks, and epidural catheter placement, providing significant pain relief and allowing time to develop a sustainable long-term treatment strategy.

This report highlights the severe impact of erythromelalgia in pediatric patients and the importance of a multimodal approach in its management. Regional anesthesia proved effective in managing acute pain and stabilizing the patient's condition.

## Introduction

Erythromelalgia is a rare, debilitating neurovascular condition characterized by episodic burning pain, erythema, and increased temperature of the extremities. The disease typically affects the feet and hands and is often triggered by warmth, exercise, or dependency of the limb, while relief is usually achieved through cooling. The condition can present in either a primary idiopathic form or as secondary to other systemic diseases such as myeloproliferative disorders, autoimmune diseases, or infections [1]. Pediatric cases are especially rare and often more challenging to manage due to the limited data available and the complexity of pain management in children [2]. The overall incidence of erythromelalgia is estimated to be between 0.25 and 1.3 per 100,000 people per year, with pediatric cases being particularly uncommon [3]. While several pharmacologic and non-pharmacologic treatments have been explored [4], there is currently no universally effective therapy. This report discusses a 14-year-old patient whose severe pain led to a suicide attempt, highlighting the effective use of regional anesthesia for acute pain management.

## Case presentation

A 14-year-old patient, undergoing treatment for tuberculosis for the past month, presented with symptoms suggestive of erythromelalgia; however, the diagnosis had not yet been established at that time. The patient's symptoms began a month and a half ago with paroxysmal, bilateral, and symmetrical painful episodes affecting both feet. These episodes were characterized by intermittent redness, a burning sensation, warmth, and swelling of the feet. Initial management included analgesic treatment with nonsteroidal anti-inflammatory drugs (NSAIDs), pregabalin, and paracetamol. Despite this, 20 days into treatment, the patient’s pain worsened, becoming constant and resistant to both pharmacological and physical measures. There was no family history of similar symptoms or related conditions.

The patient was admitted to the pediatric ward for further diagnostic evaluation and pain management. An extensive workup was conducted, including thoraco-abdominal CT scans, X-rays of the limbs, soft tissue ultrasounds, autoimmune panels, a myelogram, and a bone marrow biopsy. This comprehensive evaluation was non-conclusive, ruling out any secondary causes, leading to the diagnosis of primary erythromelalgia. As for pain management, an analgesic regimen consisting of paracetamol, NSAIDs, pregabalin, and morphine was administered. However, due to the persistence and escalation of pain, which had become unbearable, the patient attempted suicide, necessitating admission to the intensive care unit (ICU).

**Figure 1:**
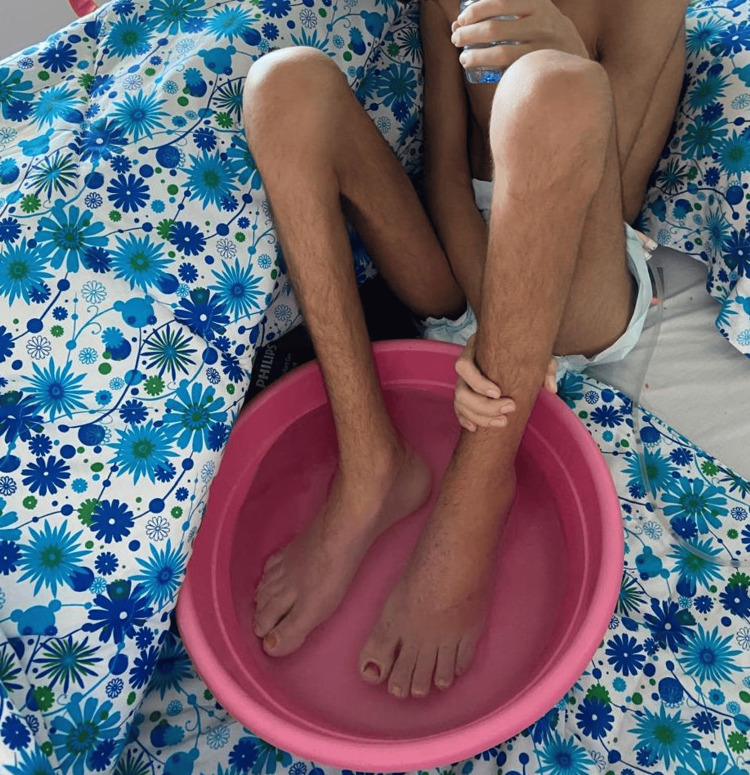
The image shows the patient upon ICU admission, crouching with his swollen, erythematous feet submerged in a bucket of water, a measure to relieve his severe pain.

Upon ICU admission, the patient was in severe pain, with a Visual Analog Scale (VAS) score of 10, exhibiting extreme distress by crouching and repeatedly striking his feet and submerging them in a bucket of water for relief (Figure 1). Dermatological examination revealed symmetrical, bilateral swelling of both feet, confined to the ankles, erythematous, and warm to touch. There were signs consistent with prolonged water immersion, but no ulcerations or necrosis were observed.

Due to the severity of the pain and the patient's hysterical state at admission, sedation was initiated using fentanyl and midazolam infusion. An analgesic protocol following the Mayo Clinic's proposed steps (including topical therapy, aspirin, gabapentin, antiepileptics, etc.) was implemented, with discontinuation of NSAIDs and opioids. Among the treatments administered, carbamazepine proved to be the most effective for our patient, leading to a significant reduction in pain levels. The patient specifically requested taking carbamazepine tablets, as they provided the most relief. Despite this improvement, the patient continued to experience nocturnal pain crises.

To address the persistent painful episodes, bilateral sciatic nerve blocks with 0.5% bupivacaine were performed, providing effective analgesia for 12 hours. This allowed the patient to sleep uninterrupted for more than eight hours for the first time in over a month. Given the ongoing pain crises, an epidural catheter was placed at the L4-L5 level, utilizing a combination of bupivacaine and fentanyl. The perimedullary anesthesia effectively managed the patient's pain while allowing time to identify an appropriate long-term treatment regimen. This approach enabled the patient to be discharged home after maintaining adequate pain control for 10 days.

The patient returned one month later with a recurrence of painful crises. An epidural analgesia was administered upon admission and was removed after five days.

Upon review five months later, the patient's symptoms had significantly improved. He was able to walk without assistance and resume his daily activities, including attending school. The pain episodes had become less frequent and less severe, allowing him to enjoy a more normal childhood routine.

## Discussion

The pathophysiology of erythromelalgia remains elusive, with current evidence suggesting a combination of vascular and neuropathic mechanisms. It is believed that dysfunction in the peripheral vasculature and abnormalities in small nerve fibers contribute to the paradoxical presentation of hyperemia alongside neural hyperexcitability, resulting in the hallmark symptoms of severe burning pain, redness, and warmth in affected extremities. Some studies have identified mutations in the SCN9A gene, which encodes the Nav1.7 sodium channel, as a significant contributor to the condition's pathogenesis. These mutations lead to a gain of function in sodium channels, causing hyperexcitability of nociceptive nerve fibers and contributing to the intense pain experienced by patients [5]. Pediatric cases, such as the one presented here, are especially challenging due to the severity of symptoms and limited data on effective management strategies.

The absence of a universally effective treatment for erythromelalgia emphasizes the need for a multimodal and multidisciplinary approach. The Mayo Clinic's proposed stepwise approach to treatment involves escalating from topical therapies and aspirin to more systemic treatments like gabapentin, carbamazepine, and other antiepileptics, with invasive procedures considered only when necessary [2]. Despite such structured management plans, pediatric patients often experience refractory pain that significantly impacts their quality of life, as was evident in this case.

In our case, initial management with NSAIDs, pregabalin, and paracetamol was ineffective. The escalation to opioid use also proved insufficient and brought concerns about long-term adverse effects, consistent with current literature discouraging prolonged opioid use in managing erythromelalgia [6]. Instead, carbamazepine emerged as the most effective pharmacologic agent for our patient, significantly reducing pain levels and improving pain management.

The use of regional anesthesia, specifically peripheral nerve blocks and epidural anesthesia, played a crucial role in managing this patient’s pain. Bilateral sciatic nerve blocks provided immediate and significant pain relief, allowing the patient to experience uninterrupted sleep for the first time in weeks. This intervention was pivotal in managing acute pain crises and preventing further psychological distress, which was critical given the patient’s recent suicide attempt. The subsequent use of an epidural catheter further stabilized pain control, allowing time to refine the pharmacological management strategy. This approach aligns with other reports suggesting regional anesthesia as a valuable tool for managing refractory pain in erythromelalgia [7,8].

By blocking nociceptive pathways at the spinal level, epidural anesthesia not only provides pain relief but also reduces peripheral neural hyperexcitability and central sensitization, which are thought to contribute to the chronic pain experienced by erythromelalgia patients.

Moreover, the use of regional anesthesia in this case provided a crucial therapeutic window, allowing time to identify and initiate a more sustainable long-term pharmacologic regimen. This approach underscores the potential of regional anesthesia not only as a means of pain relief but also as a strategic tool in the broader management plan for erythromelalgia, offering temporary respite while longer-term therapies are optimized.

## Conclusions

This case highlights the severe impact of erythromelalgia on pediatric patients, which can lead to suicide attempts, and underscores the challenges of managing this debilitating condition. A multimodal approach, incorporating both pharmacological management and regional anesthesia, offers hope in managing the complex pain associated with erythromelalgia. Regional anesthesia, in particular, provides immediate and effective pain relief during crises, enabling the stabilization of the patient's condition and allowing time to explore more effective long-term treatments. Further studies are needed to establish standardized treatment protocols and to explore the long-term outcomes of regional anesthesia in pediatric erythromelalgia.
